# Defining the functional states of Th17 cells

**DOI:** 10.12688/f1000research.6116.1

**Published:** 2015-05-28

**Authors:** Youjin Lee, Vijay Kuchroo

**Affiliations:** 1 Evergrande Center for Immumnologic Diseases, Harvard Medical School and Brigham and Women's Hospital, Boston, MA, 02115, USA; 2 Genomic and Biotechnology Section, Faculty of Science, King Abdulaziz University, Jeddah, 21589, Saudi Arabia

**Keywords:** Th17, T helper cells, inflammation, cytokine signaling

## Abstract

The molecular mechanisms governing T helper (Th) cell differentiation and function have revealed a complex network of transcriptional and protein regulators. Cytokines not only initiate the differentiation of CD4 Th cells into subsets but also influence the identity, plasticity and effector function of a T cell. Of the subsets, Th17 cells, named for producing interleukin 17 (IL-17) as their signature cytokine, secrete a cohort of other cytokines, including IL-22, IL-21, IL-10, IL-9, IFNγ, and GM-CSF.  In recent years, Th17 cells have emerged as key players in host defense against both extracellular pathogens and fungal infections, but they have also been implicated as one of the main drivers in the pathogenesis of autoimmunity, likely mediated in part by the cytokines that they produce. Advances in high throughput genomic sequencing have revealed unexpected heterogeneity in Th17 cells and, as a consequence, may have tremendous impact on our understanding of their functional diversity. The assortment in gene expression may also identify different functional states of Th17 cells. This review aims to understand the interplay between the cytokine regulators that drive Th17 cell differentiation and functional states in Th17 cells.

## T helper subsets and links to autoimmune inflammation

**Figure 1.**
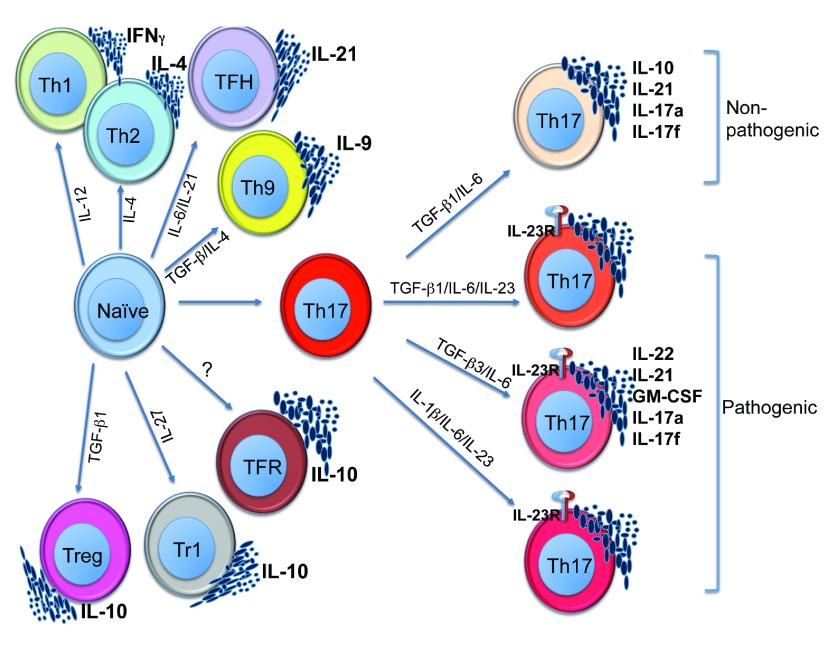
The diversity of CD4 subsets. CD4 T helper subsets identified with differentiating conditions as well as the signature cytokines they are known to produce. Th17 cells are further subtyped based on cytokine conditions that define pathogenic *versus* non-pathogenic states.

CD4 T cells are essential architects of host immune defense against pathogens ^1, 2^. Collectively, their effector function is mediated in part by a compilation of cytokines that directs differentiation, migration, homeostasis, regulation, and inflammation. Initially, CD4 T helper (Th) cells were grouped into two distinct subsets defined by production of unique cytokines: type 1 helper T cells (Th1) produce IFNγ as their signature cytokine and mediate immune responses against intracellular pathogens, and type 2 helper T cells (Th2) secrete interleukin (IL)-4, IL-5 and IL-13 and drive immune responses against extracellular pathogens, like parasites ^3^. In recent years, the number of unique subsets has grown to include IL-9-producing Th9, follicular T helper cells (Tfh) and IL-17-producing Th17, as well as three subsets of T cells that regulate immune responses, including Type 1 regulatory cells (Tr1), follicular T regulatory cells (TfR) and T regulatory cell (Tregs) ( Figure 1) ^4– 6^. Each of the effector subsets is not only critical for orchestrating a proper immune response against specific pathogens but is also a major contributor in the pathogenesis of a number of autoimmune inflammatory diseases ^7^.

For a number of years, IL-12-induced Th1 cells were thought to be the main drivers of autoimmunity, based on the findings that IFNγ-secreting CD4 T cells were frequently found at the site of inflammation and treatment with IFNγ led to exacerbated disease in multiple sclerosis patients ^8, 9^. IL-12 is a heterodimeric cytokine composed of two subunits, IL-12p35 and IL-12p40, and is a critical factor for the differentiation of Th1 cells ^10, 11^. CD4 T cells express a heterodimeric IL-12 receptor (IL-12R) composed of IL-12Rβ1 and IL-12Rβ2 subunits ^12, 13^. Upon exposure to IL-12, the master transcription factor *Tbx21* is induced, which transactivates IFNγ and the cells differentiate into Th1 cells ^14, 15^. The importance of Th1 cells in autoimmune diseases was further supported by findings that protection from experimental autoimmune encephalomyelitis (EAE), an animal model of multiple sclerosis, was observed upon neutralization with anti-IL-12p40 or in IL-12p40 ^–/–^ mice ^16, 17^. However, it became clear that Th1 cells may not be the exclusive drivers for autoimmunity when it was discovered that mice lacking critical components of the Th1 differentiation pathway, such as IFNγ, IFNγR, IL-12Rβ2, and IL-12p35, were highly susceptible to EAE, suggesting that Th1 cells may even be protective in autoimmune diseases ^18– 22^.

## Discovery of IL-23- and Th17-associated pathogenic inflammation

In the late 1990s, a novel cytokine called IL-23 that belongs to the IL-12 family of cytokines was discovered ^23^. Interestingly, similar to the functional IL-12 cytokine, IL-23 had an IL-23 p19 subunit, which combined with the IL-12 p40 subunit of IL-12, to develop a functional heterodimeric cytokine ^24^. Loss of either IL-23 p19 or IL-12 p40 chains made mice highly resistant to the development of EAE and other autoimmune diseases, suggesting that IL-23 is a cytokine critical for development of autoimmunity ^17, 25, 26^. However, unlike IL-12, IL-23 did not induce IFNγ production from naïve CD4 T cells ^24, 27^, but it was suggested that IL-23 may be critical for the generation of IL-17-producing Th17 cells. A series of *in vitro* studies showed that IL-23 could not induce differentiation of naïve T cells into IL-17-producing Th17 cells. In fact, it was discovered that the receptor for IL-23 was not even expressed on naïve CD4 T cells, suggesting that other cytokines besides IL-23 may be critical for the generation of Th17 cells ^28– 30^. In fact, we ^31^ and others ^32, 33^ showed that Th17 cells are differentiated in the presence of TGF-β1 and IL-6, which resulted in the induction of a unique master transcription factor called RORγt. While IL-23 was not required for the differentiation of Th17 cells, it was revealed to be a critical factor for stabilization of the Th17 phenotype and in evoking pathogenic phenotype in Th17 cells. With ensuing studies it became clear that IL-23, not IL-12, was the critical cytokine for driving autoimmune inflammation. IL-23p19 ^–/–^, IL-12p40 ^–/–^ and IL-23R ^–/–^ mice ^17, 25, 26^ were completely protected from developing a number of murine models of autoimmune diseases including EAE, psoriasis, and colitis. Consistently, Genome Wide Association Scans have reported a strong genetic linkage to single nucleotide polymorphisms (SNP) in IL-23 or IL-23R, with increased susceptibility to several human autoimmune diseases ^34– 40^. However, the clearest role of Th17 cells in human autoimmune diseases was supported by clinical studies where neutralization of IL-17 by an anti-IL-17 antibody (Secukimumab) resulted in clinically beneficial results in a number of human autoimmune diseases, including psoriasis, ankylosing spondylitis, and multiple sclerosis ^41– 45^.

## Heterogeneity within the Th17 subset

Although Th17 cells have become synonymous with autoimmune tissue inflammation, it is now clear that not all Th17 cells are pathogenic or induce tissue inflammation ^46^. In human inflammatory bowel diseases (IBDs), neutralization of IL-17 or blockade of IL-17 receptor (IL-17Ra) resulted in disease exacerbation, suggesting a possible protective role by Th17 cells ^47^. IL-17-producing T cells that line the gut mucosa do not induce inflammation but have been shown to be necessary to maintain the barrier function of the gut ^48^. Commensal bacteria in the gut may play a critical role in the generation of Th17 cells in the lamina propria and, indeed, there is an absence of IL-17-producing cells in the lamina propria of the small intestines in germ-free mice ^49, 50^. There is also evidence suggesting that IL-17 is required to prevent pathologic gut inflammation in a CD4 T cell-mediated transfer model of colitis, as cells lacking the capacity to produce IL-17, or the lack of IL-17R in recipient mice, resulted in exacerbated colitis ^51, 52^. These studies alluded to a rather novel concept: that Th17 cells are not uniform in function. In fact, we ^53^ and others ^54, 55^ have shown that Th17 cells come in two flavors: one in which they cause pathogenic tissue inflammation and autoimmune disease and the other that is non-pathogenic, in that they fail to provoke autoimmunity, especially in murine T cell models of inflammatory disease ( Figure 1) ^53– 55^. Th17 cells differentiated in the presence of TGF-β1 and IL-6 ^56, 57^ co-produce IL-17 with IL-10, do not induce tissue inflammation, and in fact may inhibit autoimmune inflammation, and thus are characterized as “non-pathogenic” Th17 cells ^55^. However, upon exposure to IL-23, a “switch” occurs in the Th17 cell transcriptome, which not only allows for stabilization of the Th17 phenotype but also converts non-pathogenic Th17 cells to become pathogenic ^53, 58^. These IL-23 experienced Th17 cells have been shown to promote destructive inflammation in numerous T cell-dependent murine models of autoimmunity ^53, 58^. IL-23 inhibits IL-10 production and instead promotes secretion of IL-22 and GM-CSF, suggesting that IL-23 drives the development of Th17 cells with unique functional properties ^59– 61^. This raises an important question: how does IL-23 induce pathogenicity in Th17 cells? Our studies revealed that IL-23 mediates important changes in the transcriptome of differentiating Th17 cells ^53^. Besides the induction of a number of unique transcription factors, IL-23 induces TGF-β3 production in developing Th17 cells ^53^. We showed that TGF-β3 together with IL-6 *in vitro* induces differentiation of pathogenic Th17 cells, without any need for further exposure to IL-23 ^53^. Similarly, John O’Shea ^54^ and Chen Dong’s ^62^ groups showed that naïve T cells exposed to IL-1β, IL-6 and IL-23 could induce Th17 cells that were highly pathogenic. Thus, by varying the cytokine cocktails *in vitro*, both pathogenic and non-pathogenic Th17 cells can be generated. Based on these observations, we undertook a systematic transcriptome analysis of Th17 cells in order to develop a novel gene signature that functionally distinguishes Th17 subsets.

## Transcriptional gene signatures for pathogenic Th17 cells

When we compared the gene expression profiles of all known possible *in vitro* differentiation combinations that induce pathogenic and non-pathogenic Th17 cells, we found 434 genes that were differentially expressed between these different Th17 subtypes ^53^. Of the 434 genes, 233 genes were differentially expressed between highly pathogenic and non-pathogenic Th17 cells ^53^. Based on the biological function, we identified a representative subset of 23 genes that was highly suggestive of driving pathogenicity ^53^. Pathogenic Th17 cells induced expression of various effector molecules that have been shown to be pro-inflammatory, such as *Cxcl3, Ccl4, Ccl5, Csf2* (GM-CSF) *, Il3* (associated with *Csf2*), *Il22, Gzmb* (Granzyme B) and, interestingly, transcription factors that are associated with the Th1 phenotype such as *Tbx21* (Tbet) and *Stat4* ^53^. Conversely, non-pathogenic Th17 cells revealed a gene signature that included molecules associated with regulation, such as *Il10* and transcription factors that regulate IL-10 production, such as *Ahr* and *Maf* in addition to *Ikzf3* (Aiolos) ^53^. In addition, non-pathogenic Th17 cells express *Il1rn* (IL-1R antagonist) which might antagonize functions of IL-1 in differentiating Th17 cells into a pathogenic phenotype ^53^. Based on the comparative gene expression profiles between pathogenic and non-pathogenic Th17 cells, our group identified a gene signature that may confer pathogenic phenotype to Th17 cells ^53^.

The dichotomous nature of Th17 cells may not be a mere *in vitro* cytokine artifact but may have occurred naturally as a consequence of evolutionary pressures to defend against different types of pathogens. Federica Sallusto’s group was first to show that human Th17 cells producing IL-10 in conjunction with IL-17 have specificity for *Staphylococcus aureus* infection ^63^. Conversely, Th17 cells that do not produce IL-10, but instead produce IFNγ with IL-17, have specificity for *Candida albicans* infection, suggesting that, evolutionarily, Th17 cells may have diverged to acquire different cytokine profiles, to become more adept in defense against specific pathogens ^63^. This is in line with clinical observations with immune-deficient patients, where the loss of transcription factor Stat3, which inhibits development of all Th17 cells, results in hyper IgE syndrome and the patients develop rampant *C. albicans* and *S. aureus* infections ^64^. Thus, based on our study, we’ve uncovered an interesting overlap in the gene expression profiles of Th17 cells specific for *C. albicans* or *S. aureus* in humans with pathogenic *versus* non-pathogenic Th17 cells in mice. The gene expression profile revealing an IL-17/IFNγ signature which was specific for *C. albicans* in humans had similarities to more pathogenic pro-inflammatory murine Th17 cells which cause severe EAE. Conversely, the gene profile for IL-17/IL-10-producing Th17 cells specific for *S. aureus* were comparable to a more non-pathogenic, regulatory gene signature ^53, 54^. This was highly suggestive of how evolutionary pressures have fine-tuned different effector cells for clearing different types of pathogens and utilized the same cells for the induction of tissue inflammation or to mediate tissue protection, albeit with small changes in the transcriptome.

## Challenges in understanding the functional outcome of Th17 heterogeneity

It has become clear in recent years that Th17 cells may have divergent functions ^53^. We are just beginning to understand the functional consequences of this extensive heterogeneity of Th17 cells ^65^. Though gene expression profiling has endowed us with the ability to identify a signature that distinguishes pathogenic from non-pathogenic Th17 cells ^53^, we do not know how these cells are naturally derived *in vivo* or what their function is in mediating tissue homeostasis, effector function, inflammation or cancer. For example, do these pathogenic or non-pathogenic Th17 cells develop simultaneously during differentiation in the lymphoid tissue or is there plasticity in the development of Th17 cells such that they can inter-convert based on the environmental cues they receive? Or perhaps there is a sequential development: do non-pathogenic Th17 cells convert into pathogenic Th17 cells during the course of maturation or differentiation? Also, given that the location of the IL-17 producing cells in the peripheral tissue is critical in dictating their function, this raises the issue of how much the peripheral tissue microenvironment alters the developmental programming of Th17 cells. Much remains to be understood in terms of how and why Th17 cells retain heterogeneity and how it influences their functional states.

In recent years, examination of heterogeneity at a single-cell resolution has become possible by high throughput single-cell RNA sequencing of whole genomes and transcriptomes ^66, 67^. Single-cell RNA sequencing allows for profiling and characterization of expression variability on a genomic scale, which provides us with the ability to correlate this genomic heterogeneity with functional differences in Th17 cells ^68, 69^. In fact, single-cell RNA sequencing of Th17 cells obtained from different tissues and lymphoid organs is allowing identification of novel regulators of functional states (pathogenic *versus* non-pathogenic) of Th17 cells (unpublished observation from our lab). Transcriptomic analysis of T cells in the secondary lymphoid organs following activation does provide valuable clues into the differentiation state acquired by the T cells, but it does not identify the functional state that may be attained by Th17 cells upon arrival into the tissue niche. The functional states (pathogenic/non-pathogenic) of the Th17 cells may be partly dependent on the cytokine milieu and tissue microenvironment to which the cells migrate in order to mediate effector functions. Utilizing the pathogenicity gene signature derived from our earlier studies ^53^ as one of the definable parameters used to analyze single-cell sequence data, our lab has discovered that the functional states of Th17 cells may be in constant flux as the T cells mediate tissue inflammation (unpublished observation). Uncovering key regulators that control effector functions of Th17 cells may permit novel treatment approaches for therapeutically inhibiting inflammation without affecting the protective functions of Th17 cells.

However, assigning function to these novel regulators will require genetic manipulation undertaken at a large scale. Unfortunately, the only way to confirm the function of a gene is through the use of knockout mice or genetic knockdowns in the cells and disease models ^65, 70^. The use of viral vectors or transfection-based si-RNA delivery was not effective in this endeavor, due to the changes in either the differentiation or cell viability induced by these manipulations ^71, 72^. Also, to generate a knockout mouse of every novel regulator identified at a single-cell level is an impossible undertaking. To bypass these obvious limitations, our lab in collaboration with Hongkun Park’s lab has developed a novel system of silicon nanowire perturbations where newly discovered candidate genes can be knocked-down at a large scale, which has improved the process of functional validation ^65^. Silicon nanowire perturbation allows for the delivery of siRNA effectively and efficiently into native T cells without the burden of activation or differentiation ^65, 73, 74^.

## The future

Armed with next-generation sequencing and silicon nanowire knockdowns, the pathogenic potential of subpopulations within Th17 cells can be revealed and novel regulators that may drive functional heterogeneity can be effectively established. Understanding the epigenetic and transcriptional controls of various functional states of Th17 cells will undoubtedly reveal new treatment paradigms for autoimmune diseases as well as give us deeper insight into the complex network that drives inflammatory *versus* tissue-protective functions of Th17 cells.
