# Supplementary material for: Defining the functional states of Th17 cells
Source: F1000Res. 2015 May 28;4(F1000 Faculty Rev):132. [Version 1] doi: 10.12688/f1000research.6116.1 (PMC4797958; doi:10.12688/f1000research.6116.1)
Supplement: Peer review history

# Peer review: Referee response for version 1

**DOI:** 10.5256/f1000research.6550.r8801

**Published:** 2015-05-28

**Authors:** Dan R. Littman

**Affiliations:** 1 New York University School of Medicine, New York, USA

I have read this submission. I believe that I have an appropriate level of expertise to confirm that it is of an acceptable scientific standard.

# Peer review: Referee response for version 1

**DOI:** 10.5256/f1000research.6550.r8800

**Published:** 2015-05-28

**Authors:** Jagadeesh Bayry

**Affiliations:** 1 French National Institute of Health and Research, Paris, France

I have read this submission. I believe that I have an appropriate level of expertise to confirm that it is of an acceptable scientific standard.

# Peer review: Referee response for version 1

**DOI:** 10.5256/f1000research.6550.r8799

**Published:** 2015-05-28

**Authors:** Richard A. Flavell, Nicola Gagliani

**Affiliations:** 1 Yale School of Medicine, Connecticut, USA

We have read this submission. We believe that we have an appropriate level of expertise to confirm that it is of an acceptable scientific standard.
